# Innovative Alkanediol-Based Eutectic Solvents for Extracting/Pre-Formulating Dermatologically Valuable Free Fatty Acids from *Spirulina* and *Porphyridium* Cakes

**DOI:** 10.3390/md22060281

**Published:** 2024-06-15

**Authors:** Laura Wils, Mervé Yagmur, Nicolas Bellin, Myriam Phelippe, Alia Chevalley, Charles Bodet, Leslie Boudesocque-Delaye

**Affiliations:** 1 UR 7502 SIMBA, Faculté de Pharmacie, Université de Tours, 31 Avenue Monge, 37200 Tours, France; 2 Laboratoire Inflammation Tissus Epithéliaux et Cytokines (LITEC), Université de Poitiers, 86073 Poitiers, Cedex 9, Francecharles.bodet@univ-poitiers.fr (C.B.); 3 Aqua Eco Culture, 7 Rue d’Armor Maroué, 22400 Lamballe, France

**Keywords:** spirulina, *Porphyridium cruentum*, biorefinery, eutectic solvents, free fatty acids, skin microbiota, alkanediols

## Abstract

The growing demand for phycobiliproteins from microalgae generates a significant volume of by-products, such as extraction cakes. These cakes are enriched with products of interest for the cosmetics market, namely free fatty acids, particularly polyunsaturated (PUFA). In this work, two cakes, one of spirulina and one of *Porphyridium cruentum*, were valorized using innovative natural hydrophobic deep eutectic solvents (NaDES) based on alkanediols. The most promising NaDES, as determined by physicochemical properties and screening, are mixtures of alkanediols and fatty acids. These include the mixtures of 1,3-propanediol and octanoic acid (1:5, mol/mol) and 1,3-propanediol and octanoic and decanoic acid (1:3:1, mol/mol). Two extractive processes were implemented: ultrasound-assisted extraction and an innovative mechanical process involving dual asymmetric centrifugation. The second process resulted in the production of extracts significantly enriched in PUFA, ranging from 65 to 220 mg/g dry matter with the two cakes. The extracts and NaDES demonstrated good safety with respect to epidermal keratinocyte viability (>80% at 200 µg/mL). The study of their impact on commensal and pathogenic cutaneous bacteria demonstrated significant effects on the viability of *Staphylococcus aureus* and *Staphylococcus epidermidis* (>50% decrease at 200 µg/mL) while preserving *Corynebacterium xerosis* and *Cutibacterium acnes*. These results highlight the potential of valorizing these co-products using alkanediol-based NaDES, in a strategy combining an active vector (NaDES) and a growth regulator extract, for the management of cutaneous dysbiosis involving staphylococci.

## 1. Introduction

Microalgae represent a unique sustainable resource of biomolecules for several industrial sectors, including the cosmetics and food industries. Microalgae can produce numerous types of biomolecules, such as proteins, pigments (phycobiliprotein, carotenoids, chlorophylls), polysaccharides, and lipids, with particular emphasis on their abundance of polyunsaturated fatty acids (PUFA) [1].

Microalgae were usually used as a powder or a global extract, targeting one class of metabolite for one defined species. For example, spirulina (*Arthrospira platensis*) and *Porphyridium cruentum* were extracted for their high content of phycobiliproteins (phycocyanin and phycoerythrin, respectively) [1]. Phycobiliproteins (PBP) are highly promising metabolites exhibiting antioxidant, anti-cancer, and anti-inflammatory activity [2,3,4]. The growing demand for phycobiliprotein-concentrated products in the food market led to the extraction of increasing amounts of microalgal biomass, using water as the main solvent [4]. By-products generated were usually used as is, even if other high value metabolites like free fatty acids (FFA) could be still valorized. Spirulina is known to present a wide profile of FFA, especially rich in polyunsaturated fatty acids (PUFA) [5]. *P. cruentum* exhibits a more limited FFA profile, generally characterized by a high amount of long-chain PUFA, especially arachidonic (C20:4) and eicosapentanoic acids (C20:5) [6].

In fact, FFA were potential new topic antibiotics, as they were known as physiological skin microbiota regulators [7,8]. One major possible valorization of microalgae FFA is in the cosmetics market to produce active ingredients to regulate the skin microbiome. In fact, unbalanced skin microbiota is linked to various skin disorders like dryness, eczema, or atopic dermatitis (AD) [9,10,11]. Especially, a predominance of *Staphylococcus aureus* was reported in lesional skin of patients suffering from AD [9,10]. One alternative strategy to antibiotic treatment is the prevention of *S. aureus* proliferation by topical addition of a moisturizing agent and/or a physiological microbiome regulator like FFA [11,12]. In this case, PUFA were highlighted as the main class of interest exhibiting both microbiota regulation and moisturizing effect [7,12].

With the growing demand for PBP-enriched products for the food and cosmetics markets, an increasing amount of PBP extraction by-products is generated each year, which could be considered an opportunity for FFA valorization. FFA are generated during PBP extraction by lipidome degradation due to intense thermal and agitation treatment, which explains why they are usually considered as lipidome degradation indicators in biofuel production. FFA are still industrially extracted using toxic solvents such as chloroform/methanol or hexane/methanol mixtures, but greener alternatives were recently described like natural deep eutectic solvents (NaDES) [3,13,14]. 

From a thermodynamic point of view, NaDES refers to liquids prepared by mixing pure compounds that display significant negative deviation from ideality, owing to their eutectic point temperature being considerably lower than that of the ideal mixture [15,16]. NaDES are obtained by mixing hydrogen bond donors with a hydrogen bond acceptor using a suitable molar ratio, based on natural compounds. They are mostly composed of primary metabolites, such as organic acids, amino acids, and sugars [16]. Hydrophobic DES (HDES) were recently introduced in the literature [17,18,19,20]. Those HDES were composed of a combination of menthol or thymol with sugars, polyols, or fatty acids, or a combination of short chain fatty acids [17,18,19,20,21,22]. 

DES were intensively studied in various fields like CO_2_ capture [23], organic and medicinal chemistry [24,25,26], green batteries [27], soft materials [28,29], or analytical sample preparation [30,31]. Considering marine resources, DES were usually described as powerful solvents for microalgal biomass pre-treatment in biofuel production [32]. Recently some DES were reported as valuable solvents for algal hydrophobic metabolite extraction. Fucoxanthin and chlorophyll of a diatom, *Thalassoria andamanica*, were extracted using a set of DES based on benzyltriethylammonium and fatty acids [33]. Fucoxanthin wax was also extracted from an alga, *Tisochrysis lutea*, using NaDES based on fatty acids and terpenes, like thymol/C8 [34]. A set of HDES based on oleic acid and terpenes, like thymol or geraniol, was also screened for astaxanthin extraction from *Haematococcus pluvialis* [35]. Considering microalgae lipids, omega 3 lipids were recovered from *Nannochloropsis* using choline chloride-based DES, focusing on ester lipids and not FFA [36]. Our group has recently reported the extraction of FFA from fresh spirulina biomass using a panel of NaDES [3,13,14]. Fatty-acid-based NaDES, especially (C9/C10/C12, 3:2:1, mol/mol/mol), were reported as solvents with high capacity for FFA solubilization but were highlighted as more specific to saturated FFA [3,14]. Fatty-acid-based DES were also reported as potential antimicrobial media for drug vectorization [37]. NaDES based on menthol and alkanediols (1,3-propanediol, 1,2-octanediol) were also highlighted as selective solvents for monounsaturated FFA (MUFA) and PUFA, but with lower solubilization capacity [13].

Compared to organic solvents with low toxicity, NaDES exhibited several advantages, especially in biomass extraction. First, NaDES could be kept in the final product, avoiding the time- and energy-consuming steps of solvent removal and recycling [13]. From an environmental point of view, some NaDES have a lesser impact, especially those based on sugars or polyols like alkanediols [38]. And the safety of non-volatile solvents like NaDES is much higher than bio-based solvents like ethanol.

Indeed, to produce ready-to-use cosmetics ingredients for skin microbiota regulation, NaDES components should be in line with European and Chinese cosmetics regulations, which exclude choline chloride and most terpenes considered to be allergens [39]. Combining FFA and natural skin microbiota regulators with antimicrobial DES based on fatty acids should result in enriched extracts that could favor the regulation of microbiota diversity and prevent *S. aureus*-associated dysbiosis, linked to skin disorders such as AD or sensitive skin. 

Ultrasound-assisted extraction (UAE) is a green technology that has been widely adopted for biomass extraction using NaDES [40,41,42]. This technology is based on the phenomenon of cavitation, which significantly improves material transfer by promoting the destruction of cell walls, particularly microalgal ones [43]. The use of UAE has already proven its worth for the extraction or pretreatment of microalgae [44,45,46] but may generate significant temperature rises that can impact the stability of heat-sensitive metabolites such as PUFA.

Dual asymmetric centrifugation (DAC) is a process originally designed for material mixing and dispersion in semi-solid materials [47,48,49]. The frictional forces generated when the centrifuge is rotated enable very rapid transfer and high friction over short operating times [49]. Our group was the first to use the DAC process for plant extraction, using a betaine- and glycerol-based NaDES to extract antioxidant metabolites. This technology enabled us to obtain extracts significantly enriched in carotenoids, thanks to the absence of a temperature rise [50]. This technology should therefore be studied to promote the extraction of carotenoids and PUFA from cakes.

In this work, we explored the design of a new class of HDES based on alkanediols to improve PUFA extraction performance. NaDES’ solubilization capacity towards carotenoids and palmitic acid was investigated. The most performant NaDES were screened on cakes of spirulina and *P. cruentum*, resulting of PBP water extraction. Two extraction processes were challenged: ultrasonic extraction and an innovative dual asymmetric centrifugation (DAC). A selection of the resulting extracts was then tested on keratinocytes and four bacterial species of skin microbiota to highlight potential benefit for skin dysbiosis prevention. 

## 2. Results and Discussion

### 2.1. Alkanediol-Based NaDES Design and Characterization

Alkanediol-based NaDES were already described in the literature, mostly combined with choline chloride, for polar metabolite extraction [51,52,53]. Some recent work reported interest in combining alkanediols with different HBA to enhance the extraction of hydrophobic metabolites like terpenes [54], and curcumin [55]. Most of the NaDES described then were either not compatible with use in cosmetics or too polar to extract FFA. Our lab has recently highlighted the potential of menthol–alkanediol combination to extract PUFA from fresh spirulina biomass [13], highlighting the potential of alkanediols for our purpose.

To design our alkanediol-based NaDES library, the Smart NaDES selection strategy recently described by our group was applied [13]. Raw materials were first selected in accordance with European and Chinese cosmetics regulations [39], to avoid the metabolites/NaDES separation step. Two alkanediols were then selected as HBD components: 1,3-prodanediol and 1,2-octanediol. To prepare a wide range of alkanediol-based NaDES, HBA with a large range of polarity were selected from glycerol to octanoic acid (Table 1).

Most of the alkanediol-based NaDES produced were easy to prepare using stirring and heating protocols at low temperatures (between 50 and 60 °C), except for NaDES containing citric acid. In general, the density was acceptable for the extraction process (between 0.88 to 1.28). Especially, NaDES based on alkanediols and fatty acids exhibited the lowest density, allowing easy transfer and handling (Table 1). During NaDES design, all ratios of alkanediols mixtures from 8:1 to 1:8 were investigated. NaDES were considered stable when the mixture remained limpid and stable after 7 days at room temperature.

The water miscibility of the resulting alkanediol-based NaDES was then investigated. In fact, microalgal cakes were media with low dry matter content. Therefore, NaDES of interest should not be miscible to water to avoid high amounts of water transfer during the extraction step, which could impact extract preservation. Only alkanediol combinations with fatty acid or menthol or proline were found to be non-miscible to water. To be considered as non-miscible to water, the NaDES should provide a clear biphasic system after mixing with a fixed volume of water, in less than one minute. Moreover, no volume change in water or NaDES should be noticed, highlighting the absence of water or NaDES component transfer.

To select the most relevant NaDES, the solubilization capacity of each NaDES was explored on two reference compounds: β-carotene to mimic carotenoids of interest in cakes, and palmitic acid to explore FFA solubilization capacity. Alkanediol-based NaDES performance was compared to well-known reference solvents: hexane/acetone (60:40, *v*/*v*) for carotenoids and ethyl acetate for palmitic acid.

**Figure 1:**
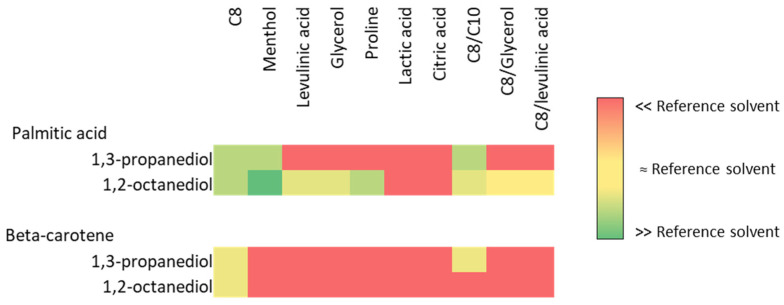
Heatmap of solubilization capacity of selected alkanediol-based NaDES towards β-carotene and palmitic acid.

Solubilization results are shown on the heatmap in Figure 1.

As we can see on the heatmap, most of the 1,2-octanediol-containing NaDES exhibited good solubilization ability of palmitic acid, except those containing lactic or citric acids. On the contrary, few 1,3-propanediol-based NaDES exhibited satisfying palmitic acid solubilization: PC8, PM, and PC8C10. This is expected due to the difference in logP of the two alkanediols (1.05 vs. 1.5) and the alkane chain length.

Considering carotenoids, only three NaDES showed performances similar to the reference hexane/acetone mixture: PC8, PC8C10, and OC8. β-carotene with high unsaturated structure could provide a good approximation of both carotenoids and PUFA behavior in NaDES.

The three NaDES combining good performance in relation to both palmitic acid or β-carotene were then selected to perform cake extraction: PC8, PC8C10, and OC8. 

### 2.2. FFA Extraction Using Hydrotrope-Based NaDES

#### 2.2.1. Cake Characterization

*A. platensis* and *P. cruentum* pretreated cakes were first characterized as our starting point. The impact of preliminary phycobiliprotein extraction on cell integrity was investigated at both macroscopic and microscopic levels. Dyes and lipid content were titrated. Pigment diversity and FFA profile were also established using HPLC and LC-ESI-MS analysis. The dry matter (DM) rate of the biomasses was measured by gravimetry (Table 2). 

**Figure 2:**
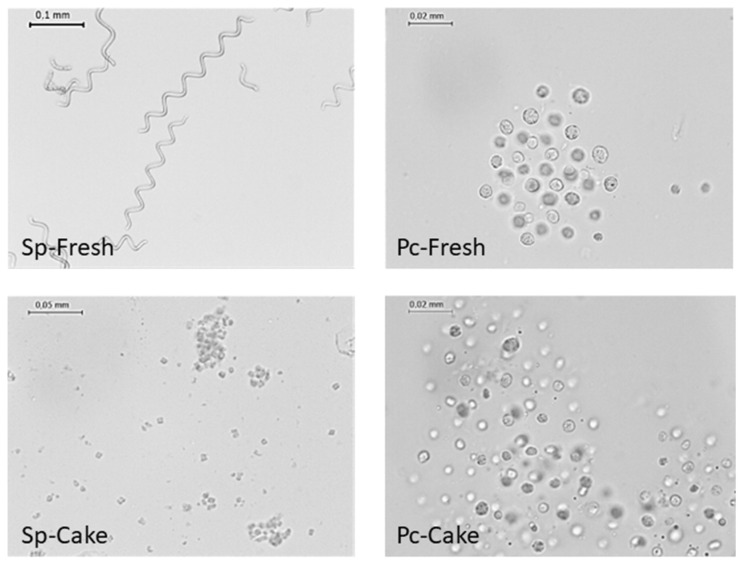
Microscopic photographs of biomasses before and after phycobilliprotein (PBP) extraction. Sp- and Pc-Fresh (biomass before extraction), Sp- and Pc-Cake (resulting by-product).

*A. platensis* was morphologically characterized by long, unbranched, spirally coiled filaments. Length and curl might vary depending on growing conditions. The fresh biomass (Sp-Fresh) exhibited a typical long spiral structure, about 250 µm long, as the dominant form (Figure 2). The *A. platensis* cake, a by-product of PBP water extraction, exhibited dramatic cellular damage with only cellular debris visible. The cells were individualized, ranging from 5 to 7.5 µm in length (Figure 2). *P. cruentum* is represented by cells of spherical shape, ranging in size from 5 to 7 µm, red to brown. As for spirulina, fresh biomass (Pc-Fresh) exhibited negligible impact on the cell wall as typical spherical structures were observed (Figure 2). Surprisingly, the preliminary extraction of PBP (Pc-cake) did not seem to impact dramatically the cell structure, but we can observe several shining and empty cells with lengths of around 4–5 µm. 

The preliminary extraction of PBP had an important impact on the cell wall in both cases, which may favor mass transfer during the lipid extraction process. Nevertheless, the impact was dramatically different between the two microalgae, linked to their very different cell-wall structures [56]. In fact, *P. cruentum* is well-known for its strong cell wall containing an important exopolysaccharide layer. This was a crucial reason to investigate the possibility of sequential extraction of PBP and FFA. Moreover, in the case of FFA, the cellular damage could be a good indicator of FFA rate, as they are recognized as lipidome degradation indicators. Targeting FFA here appeared, then, a smart strategy to upcycle these microalgae wastes.

**Figure 3:**
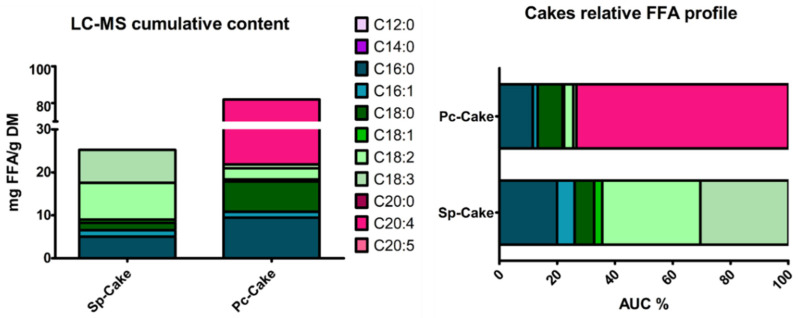
FFA profile of Sp- and Pc-Cakes in cumulative (**left**) or relative (**right**) view, according to LC-ESI-MS data.

Considering FFA profile, Sp-Cake highlighted a high ϖ6-PUFA content, as linoleic and γ-linolenic acids, in accordance with the literature (Figure 3) [5]. Lower levels of palmitic acid were observed (above 25%), differing from the usual medium rate of 40% found in the literature [5]. Sp-Cake exhibited the highest FFA rate (25 mg/g DM), as we might expect according to cell wall degradation.

Considering *P. cruentum*, the FFA profile highlighted a high level of arachidonic acid (C20:4, ϖ6), almost 80% of the total FFA (60 mg/g DM) (Figure 3). These amounts were 5-fold higher than those usually found in the literature [6]. Low levels of eicosapentaenoic acid (EPA, C20:5, ϖ3) were observed, as well as low rates of palmitic (C16:0) and linoleic acid (C18:2, ϖ6). Those FFA were usually described at higher rates [6].

Considering the pigment profiles of spirulina and *P. cruentum* cakes, as we can see in Table 1, spirulina exhibited higher chlorophyll and carotenoid content than *P. cruentum*. Sp-Cake content reached 4.4 mg/g DM for chlorophylls and 1.6 mg/g DM for carotenoids, in comparison with *P. cruentum*, which exhibited content close to 2 mg/g DM and 1 mg/d DM of chlorophylls and carotenoids respectively.

#### 2.2.2. Ultrasound-Assisted Extraction (UAE)

**Figure 4:**
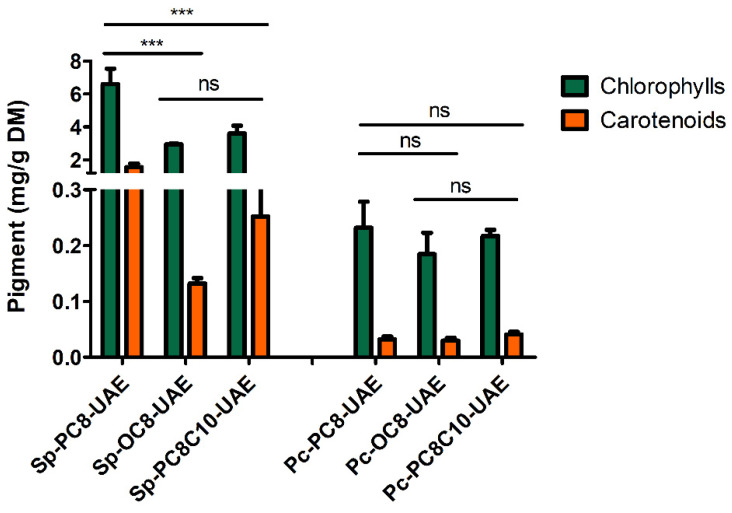
Pigment profile of NaDES cake extracts of spirulina (Sp) and *P. cruentum* (Pc). Data represent the mean ± SD. *** *p* < 0.001, ns non-significant.

UAE was explored to extract FFA using the three selected NaDES: PC8, PC8C10, and OC8. UAE was already described as a green and relevant technology to extract FFA from spirulina cakes using conventional solvents [3]. As we can see in Figure 4, the pigment profiles of all the extracts were roughly similar for *P. cruentum* and closely related for spirulina. The main difference was the lower amount of carotenoids found in the OC8 extract of spirulina cake. 

**Figure 5:**
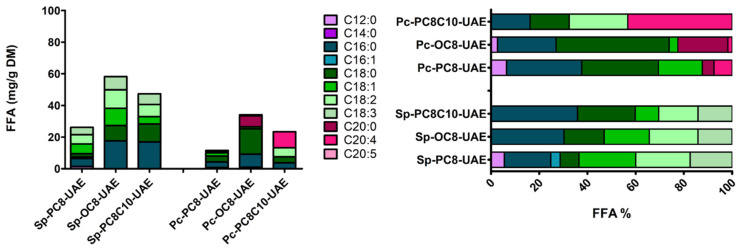
FFA profile of Sp- and Pc-Cake UAE extracts in cumulative (**left**) or relative (**right**) view, according to LC-ESI-MS data.

Considering now the FFA extraction (Figure 5), OC8 and PC8C10 exhibited the best extraction performances on both cakes, with FFA amounts of 50–60 mg/g DM spirulina and 20–30 mg/g DM *P. cruentum*. PC8 performances were twice as low with amounts of 25 mg/g DM and 15 mg/g DM for spirulina and *P. cruentum*, respectively.

The main targets of interest in the FFA pool are the unsaturated ones (MUFA and PUFA). In Figure 5, for spirulina cake extraction, it is clear that PC8 was the most performant NaDES regarding MUFA and PUFA selectivity (67% of FFA profile), followed by OC8 (53%) and PC8C10 (40%). For spirulina, PC8 appeared as the best NaDES for our purpose, combining high carotenoids and PUFA amounts, even if recovery should be improved. OC8 and PC8C10 exhibited closely related performance considering FFA. However, given the lower carotenoids levels observed in OC8, PC8 and PC8C10 were selected for spirulina extraction.

On *P. cruentum*, the most performant NaDES was PC8C10, with an unsaturated FFA rate of 67% of the FFA profile. It is important to note that this high amount was mostly composed of arachidonic acid, and that no MUFA were observed, along with a low level of linoleic acid. PC8 with lower unsaturated FFA (25%) exhibited on the contrary a more diversified profile, in accordance with the envisaged extract use. In fact, it has been shown that FFA diversity favors a better microbiota balance [7,8,12]. OC8 again exhibited the lowest selectivity towards PUFA (only 5% of FFA). Thus, considering *P. cruentum*, only PC8 was selected for further investigation.

It was interesting to note that all alkanediol-based NaDES could not provide a selective extraction between pigments and FFA. Even though chlorophylls and carotenoids were not a problem considering a cosmetic use, it would be interesting to investigate the molecular interactions between NaDES and target metabolites that could help to modulate NaDES selectivity. This is currently under investigation in our group.

#### 2.2.3. Dual Asymmetric Centrifugation (DAC) Extraction

Recently, our group explored an innovative mechanical extraction method, dual asymmetric centrifugation (DAC), to enhance flavonoid and carotenoid extraction from marigolds with NaDES [50]. This new process allowed us to work at room temperature with higher extraction recovery thanks to intense mixing between biomass and NaDES.

As PC8 and PC8C10 use in cake extraction led to interesting FFA profiles but only moderate recovery, DAC was investigated as an alternative extraction process. In fact, as the DAC process is performed at room temperature, whereas UAE generated rising temperatures during extraction (up to 50 °C), this alternative process could prevent PUFA and MUFA degradation and then increase their extraction. Extraction time (30 min) remained identical to UAE, but the rotation speed was modulated as it has a major impact on mixing intensity. Two rotation speeds were then investigated: a low one (500 rpm) and a high one (2500 rpm). 

**Figure 6:**
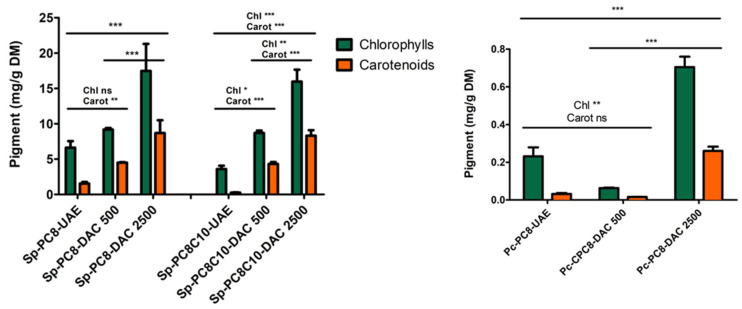
Pigment recovery from spirulina (**left**) and *P. cruentum* (**right**) cakes using DAC process and compared to UAE. Data represent the mean ± SD. * *p* < 0.05, ** *p* < 0.01, *** *p* < 0.001, ns non-significant.

As shown in Figure 6, pigment recovery increased with speed rotation for both microalgae cakes. With PC8 the carotenoid amount increased 6-fold, from 1.5 mg/g DM using UAE to 8 mg/g DM with DAC at 2500 rpm. The performance of DAC at 2500 rpm was even more impressive on *P. cruentum* (10-fold) and with PC8C10 on spirulina (25-fold). Considering FFA recovery, the use of the DAC process, particularly at 2500 rpm, dramatically improved the extraction performances for all NaDES screened and the two microalgae cakes. 

**Figure 7:**
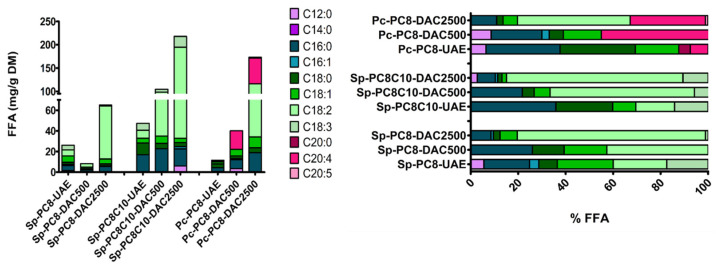
FFA profile of Sp- and Pc-Cake UAE and DAC extracts in cumulative (**left**) or relative (**right**) view, according to LC-ESI-MS data.

Considering spirulina, the FFA amount was raised from 26 mg/g DM to 65 mg/g DM with PC8 and from 47 mg/g DM to 218 mg/g DM for PC8C10 (Figure 7). It is also important to note that the FFA profile, using the DAC process, was then dominated by PUFA and MUFA (almost 90% of FFA). Linoleic acid (ϖ6 PUFA) represented 79 and 74% of the FFA fraction of the Sp-PC8-2500 and Sp-PC8C10-2500 extracts, respectively (Figure 7). This is particularly interesting for cosmetics use as linoleic acid is well known as a major inflammation and microbiota regulator for skin [7,10,12]. Comparing to previous results obtained on spirulina cakes with C9C10C12 (3:2:1, mol/mol/mol), alkanediol-based NaDES combined with DAC enhanced the recovery of PUFA in particular about 10-fold [3]. It was also interesting to note that the global extraction of FFA was impaired from 162 mg/g DM to 60 mg/g DM for PC8 but increased to more than 200 mg/d DM with PC8C10. This points in favor of new molecular interaction that selectively targets PUFA thanks to alkanediol introduction [3].

Considering *P. cruentum*, the FFA amount was raised from 11 mg/g DM to 173 mg/g DM using PC8C10. Again, the relative proportion of MUFA and FFA increased to reach almost 90% of the FFA profile, with a more diversified profile. Indeed, linoleic acid was found at an equivalent rate to arachidonic acid. This extract exhibited, then, a more interesting profile for our purpose. The DAC process at high rotation speed was an interesting option to consider to enhance FFA recovery from PBP extraction cakes, especially MUFA and PUFA. During all extraction experiments, the temperature was maintained below 35 °C, which could explain the preservation of PUFA in the extracts. Three samples were then selected to be biologically investigated: Sp-PC8-2500, Sp-PC8C10-2500, and Pc-PC8-2500.

### 2.3. Biological Evaluation for Cosmetics Use

Before considering a potential cosmetics use of our alkanediol-based NaDES extract, the harmlessness on skin and the impact on microbiota were preliminary investigated using keratinocytes, the most abundant cell type of the epidermis, and four bacterial species of the skin microbiota.

To separately highlight NaDES and metabolite effects, all experiments were performed using NaDES alone and their resulting extracts.

#### 2.3.1. Impact on Keratinocyte Viability

To investigate the safety of NaDES and their extracts for dermatological applications, the in vitro viability of HaCaT cells, an immortalized human keratinocytes line, was evaluated after a 24 h treatment using the XTT colorimetric assay, which measures cellular metabolic activity, and the lactate dehydrogenase (LDH) release assay, a marker for cell lysis. The concentrations of the compounds were set at 25, 50, 100, and 200 µg/mL, as described by Wils et al. [14]. In addition to a negative control (unstimulated cells), a DMSO control was considered as the NaDES, and extracts were diluted in DMSO. No impact of DMSO was observed at the highest concentration.

**Figure 8:**
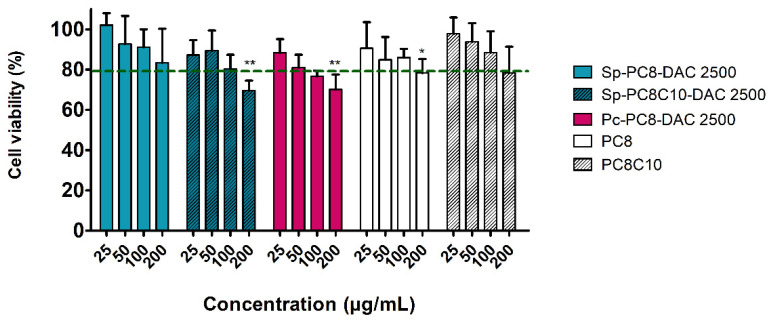
Cell viability of keratinocytes after treatment with alkanediol-based NaDES or NaDES extracts (XTT assay). Results are expressed as percentages of cell viability relative to unstimulated control cells. Data represent the mean ± SEM of four independent experiments. * *p* < 0.05, ** *p* < 0.01.

Overall, except for the Sp-PC8-2500 extract and PC8C10 NaDES, a significant decline in cell viability was observed only at the maximum concentration of 200 µg/mL in comparison to the control cells (Figure 8). Subsequently, cell viability gradually increased to 90–100% as the concentration decreased for all samples tested. Alkanediol-based NaDES and their extracts thus appear to exhibit satisfactory safety on keratinocytes, as cell viability was approximately 80% at the highest concentration. This slight toxicity is in accordance with the established cosmetic standards established by Caprin et al. [57], which permit a viability of 80% for 1% (*v*/*v*) of NaDES or extract diluted in the culture medium. This concentration range is between 8 and 20 µg/mL, depending on the density of the considered NaDES or extract. Consequently, all the samples can be considered safe, with the viability of keratinocytes only being impacted for concentrations at least 10 times higher. As there was no significant difference observed between the NaDES and the extracts, it can be concluded that the cytotoxicity is mainly due to the presence of NaDES.

To validate these findings, a second cytotoxicity test, the LDH release assay, was then carried out. The results obtained did not indicate plasma membrane permeabilization or cell death for any samples tested even at the highest concentration tested. Therefore, the cytotoxicity observed in the XTT test can be attributed to a slowing down of metabolism rather than real cytotoxicity. To ensure the cells were healthy (adherent and alive), a microscopic control was also conducted. Microscopy did not indicate any morphological abnormalities in the treated cells, confirming the absence of cytotoxicity in the literal sense.

To date, only three publications have evaluated the toxicology of NaDES on HaCaT cells. These studies found that all the hydrophilic NaDES tested did not show cytotoxicity on keratinocytes [14,57]. Regarding the cytotoxicity of hydrophobic NaDES on HaCaT, Silva et al. examined the toxicity of DES derived from menthol and fatty acids (lauric, stearic, or myristic acids) [37,58]. These DES exhibited cytotoxicity at concentrations of 12 mM, and the presence of menthol was found to be correlated with toxicity. Those data were confirmed by the study of Wils et al., which demonstrated the cytotoxicity of NaDES based on menthol, with 76% cell viability observed at 200 µg/mL. Fatty-acid-based NaDES also exhibited a cytotoxicity at high concentrations (200 µg/mL) of 57% and 56% cell viability for C8/C12 and C9/C10/C12 NaDES respectively [14]. It was then interesting to note that de alkanediol/fatty acids combination described here appeared to be safer for keratinocytes.

#### 2.3.2. Impact on Skin Microbiota Bacterial Strains

**Figure 9:**
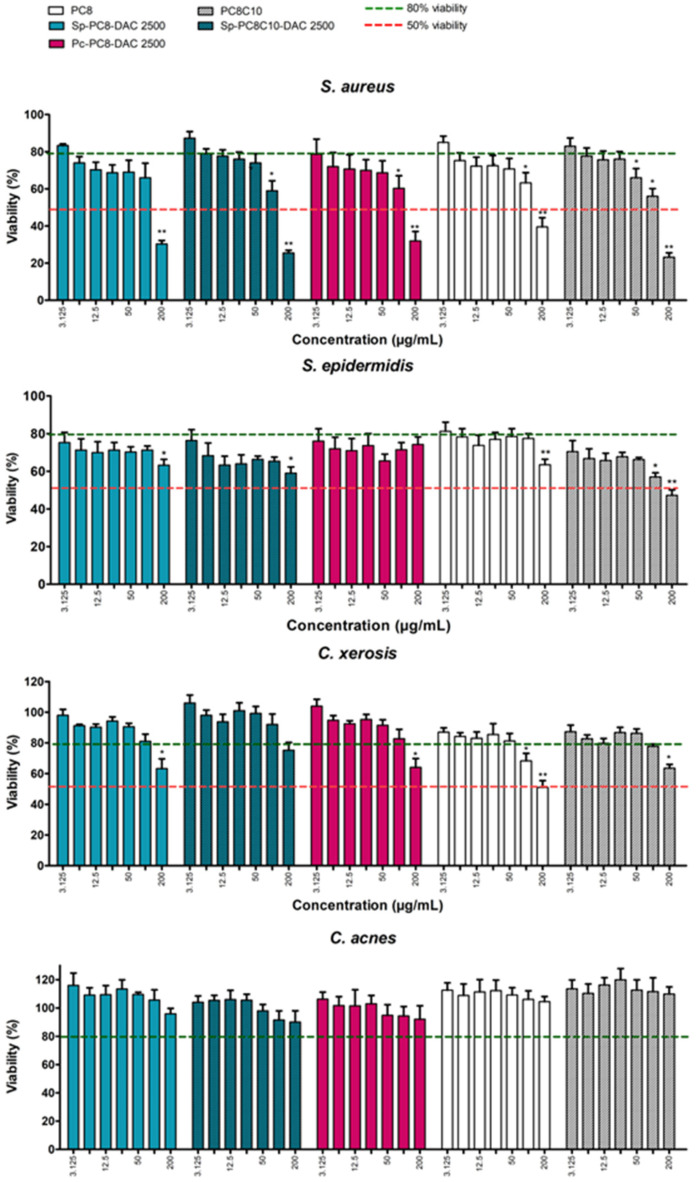
Cellular viability of bacteria after treatment with alkanediol-based NaDES or NaDES extracts (XTT assay). Results are expressed as percentage viability relative to unstimulated control cells. Data represent the mean ± SEM of four independent experiments. * *p* < 0.05, ** *p* < 0.01.

Cell viability was also assessed in bacteria from the skin microbiota using the XTT assay. The tests were conducted on four Gram-positive species: *Corynebacterium xerosis*, *Cutibacterium acnes*, *Staphylococcus epidermidis*, and *S. aureus*, the latter of which is associated with atopic dermatitis severity. The cytotoxic profiles induced by NaDES and their extracts at concentrations ranging from 200 μg/mL to 3.125 μg/mL are presented in the following figure (Figure 9).

A dose–response effect is observed in the two species of Staphylococci, whereby an increase in compound concentration is accompanied by a decline in cell viability (Figure 9). At 200 µg/mL, except for Pc-PC8 on *S. epidermidis*, all the compounds are highly deleterious, with a viability of less than 30% for *S. aureus* and 65% for *S. epidermidis*. Except for Pc-PC8 and PC8 on *S. epidermidis*, the difference from the control is also significant at 100 µg/mL for most of them, with a viability of close to 60–70%. For the other concentrations tested, viability rises slowly with increasing concentration. At the minimum concentration, 100% viability is not achieved. The antibacterial activity seems linked to the presence of NaDES, with few differences between the extracts and NaDES alone, except on *S. epidermidis*, where the NaDES have more advanced antibacterial activity than the extracts.

The other two bacterial species, *C. acnes* and *C. xerosis*, are much less impacted (Figure 9). Regarding *C. acnes*, viability is close to 90–100% for all conditions, with no significant difference between the control and the highest concentration tested. In contrast, compounds at 200 µg/mL appear to be toxic for *C. xerosis*, with a viability of 50–70%. At 100 µg/mL, viability reaches at least 80%, except for PC8, for which viability remains significantly reduced. For all concentrations and solvents combined, the cytotoxic activity of the extracts on *C. xerosis* is lower than that of the corresponding NaDES, particularly at the lowest concentrations (*p* < 0.05). This observation is important to the approach developed here, as it highlights a protective effect of the extract regarding the intrinsic toxicity of the solvent. It can be reasonably concluded that the combined effects of the extracts and NaDES would result in a slowing of the metabolism of *Staphylococci* species, which are known to be predominant in the dysbiosis of the skin microbiota associated with atopic dermatitis. The fact that the viability of commensal strains, such as *C. xerosis* or *C. acnes*, is less or not impacted can favor the skin microbiota to tend towards a rebalancing. Spirulina extracts appear to be the most promising, with this duality of effect observable from 50 µg/mL. 

Hydrophobic alkanediol-based NaDES therefore appear to have increased toxicity on Staphylococcal species compared to the commensal flora. This is consistent with the results previously published by our group on fatty-acid-based NaDES [14]. Many authors have shown that low concentrations of saturated FFA are bacteriostatic but higher concentrations cause lysis of bacteria [8,12,59]. The mechanism of toxicity of (Na)DES on bacteria remains poorly understood despite research which has intensified in recent years. Just like on eukaryotic cells, toxicity varies with the concentration and composition of DES. The authors assume that the toxic effect would be linked to the disruption of cell walls, due to the presence of delocalized charges. The toxic effect would be accentuated by the presence of an organic acid (acidic pH) and would be less marked in the presence of NaDES containing water, surely due to the dilution of the NaDES leading to lower toxicity by distention of the network hydrogen bonds [60]. Nevertheless, the antibacterial effect is an advantage for NaDES in the context of their use, particularly in the biomedical, agri-food, or cosmetics fields. In addition to potential activity on microbiota and pathogenic germs, this intrinsic activity would also make it possible to avoid the addition of controversial inputs such as antimicrobial preservatives. NaDES based on fatty acids and alkanediols, therefore, showed anti-staphylococci activity. At the same time, the extracts appear more beneficial for commensal bacteria. Hydrophobic NaDES formulations can therefore be considered as regulators of the microbiota in the case of *S. aureus*-associated dysbiosis, particularly in the context of atopic or atopic-prone skin.

**Figure 10:**
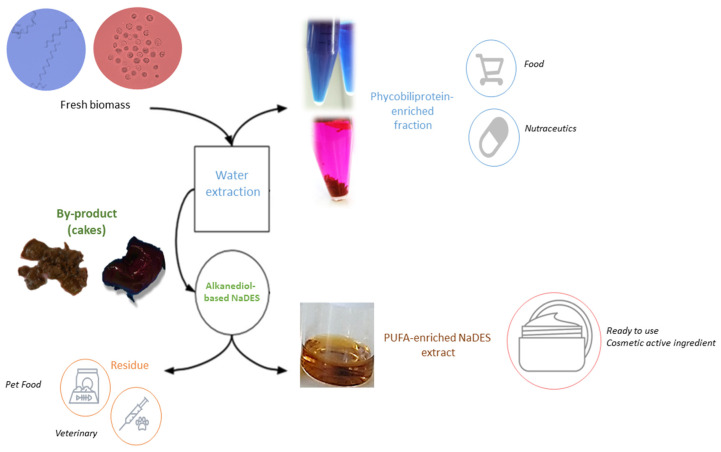
General workflow from fresh microalgal biomass to ready-to-use cosmetics ingredients.

The proposed workflow enables a true biorefinery approach for phycobiliprotein-rich microalgal strains (Figure 10). Waste is not produced throughout the value chain. PBP-enriched extracts are marketed in aqueous mediums as dietary supplements or food colorings, while extracts pre-formulated in NaDES medium are directly usable by the cosmetics industry as microbiota-regulating active ingredients. Moreover, the Smart NaDES strategy enables the direct valorization of extraction residues in the pet food market, due to the compatibility of NaDES components with the food and veterinary markets.

Those alkanediol-based NaDES could be also explored on non-polar compounds of interest like gingerol, carminic acid, or astaxanthin to enlarge their application field [34,61,62,63].

## 3. Materials and Methods

### 3.1. Raw Material

Spirulina (*Arthrospira platensis*) (harvested and extracted in July 2019) and *Porphyridium cruentum* (harvested and extracted in August 2020) cakes were kindly provided by Aqua Eco Culture (Lamballe, France). All the biomasses were stored at −20 °C. Cakes were obtained after the extraction of phycobiliproteins using the internal Aqua Eco Culture process.

#### 3.1.1. Microscopy

Cells before and after phycobilliprotein extraction were observed under a trinocular microscope LEICA L3000 and photographed using a digital camera, the LEICA EC3 (Leica Camera AG, Wetzlar, Germany). The photos were edited with LEICA’s associated software, LAS EZ version 1.6.0.

#### 3.1.2. Dry Matter Determination

Dry matter content was determined using gravimetry. Briefly, 1 g of biomass was introduced into the infrared moisture meter MB23 (Ohaus, Nänikon, Switzerland) at 80 °C until stable mass was observed. 

### 3.2. Chemicals

Ethyl acetate (EtOAc), methanol (MeOH), acetonitrile (ACN), acetone, hexane, and propan-2-ol were purchased from Carlo Erba (Val de Reuil, France). Ammonium formate, DMSO, and saturated and unsaturated fatty acids standards kit were purchased from Merck-Sigma Aldrich (Saint-Quentin Fallavier, France). Palmitic acid (99%), octanoic acid (99%), levulinic acid (98%), betain (98%), L-proline (99%), 1,3-propanediol (98%), and 1,2-octanediol (98%) were purchased from Acros Organics (Geel, Belgium). Formic acid was purchased from Fisher Scientific SAS (Illkirch, France). Nonanoic acid 98%, decanoic acid 99% and beta-carotene (99%) were purchased from Alfa Aesar (Haverhill, MA, USA). Water was purified using a Milli-Q system (Millipore Corporation, Bedford, MA, USA). LC-MS-grade formic acid was purchased from Fisher Scientific SAS (Illkirch, France). 

### 3.3. NaDES Preparation and Characterization

#### 3.3.1. NaDES Preparation

NaDES were prepared by mixing appropriate ratios of HBA and HBD (see Table 1). The mixtures were heated at 50 °C or 80 °C and stirred until colorless liquids were obtained [14].

#### 3.3.2. Density 

NaDES density in g/mL was determined by withdrawing one milliliter of NaDES with an automatic pipette set for viscous liquids (AutoRep Rainin, Brand, Wertheim, Germany) and weighing it on a precision balance (Pioneer, Ohaus, Nänikon, Switzerland). All measures were performed in triplicate.

#### 3.3.3. Water Miscibility

An amount of 1 mL of water was added to 1 g of NaDES in a graduated hemolysis tube. Phases were mixed at 30 °C under magnetic stirring for 1 h. The samples were then visually analyzed for volume check of each phase. NaDES were considered non-miscible with water if the NaDES phase volume exhibited less than 20% variation.

#### 3.3.4. Palmitic Acid and Beta-Carotene Solubilization Ability

For palmitic acid, 500 mg of solvent was added to a 1.5 mL Eppendorf tube, then the appropriate amount of palmitic acid was added, according to the desired concentrations: 20 mg/mL, 50 mg/mL, 100 mg/mL, and 200 g/mL. Solubilization was ensured by a 30-min ultrasonic cycle (RK-100H, Bandelin Electronic, Berlin, Germany). Solubilization was visually checked: a clear sample was considered soluble; a non-homogeneous sample or precipitate was considered to be at the saturation limit. NaDES performances were compared to EtOAc, the biosourced reference solvent [3]. 

For beta-carotene, 500 mg of solvent was added to a 1.5 mL Eppendorf tube, then the appropriate amount of beta-carotene was added, according to the desired concentrations: 1.0 mg/mL, 2.5 mg/mL, and 5 mg/mL. Solubilization was ensured by a 30-min ultrasonic cycle (RK-100H, Bandelin Electronic, Berlin, Germany). Solubilization was visually checked: a clear sample was considered soluble; a non-homogeneous sample or precipitate was considered to be at the saturation limit. NaDES performances were compared to hexane:acetone (60:40, *v*/*v*), the reference solvent [3].

### 3.4. Extraction Process

#### 3.4.1. NaDES Screening and UAE

Biomass was extracted with NaDES (or reference solvent) using protocol described in [3], without modifications. Briefly, biomass was extracted with NaDES or heptane using ultrasound-assisted extraction (UAE) for 30 min with biomass/solvent ratios of 1/6, *w*/*w* and 1/20, *w*/*w*, respectively. The resulting extracts were centrifuged at 16,200× *g* (Rotanta 460 R, Hettich, Kirchlengern, Germany) for 20 min. The supernatant was collected and analyzed.

#### 3.4.2. Dual Asymmetric Centrifuge Extraction

Spirulina and *P. cruentum* cakes were extracted using an asymmetric Hauschield Speedmixer DAC 150 (MP2E Solutions, Fontenay aux Roses, France) with the following parameters. C8/Prop (5:1, mol/mol) and C8/C10/Prop (3:1:1, mol/mol/mol) (3 g) and the cakes (0.6 g) were introduced in appropriate vials. Samples were then extracted at 500 or 2500 rpm for 30 min [50].

The resulting extracts were then recovered after centrifugation at 16,200× *g* (Rotanta 460R, Hettich, Sarreguemines, France).

### 3.5. Analytical Protocols

All extracts and the initial biomass were analyzed in terms of pigment content, lipid content, and FFA and pigment profiles. Spirulina extracts were named “Sp-X” and *P. cruentum* were named “Pc-X” according to the solvent used. For example, the resulting extract of spirulina obtained with 1,3-propanediol:C8 was named “Sp-PC8”.

#### 3.5.1. Pigment Content

Initial biomasses were characterized in terms of global dye content. Biomasses were extracted by water under magnetic agitation for 2 h with a biomass dry matter/solvent ratio of 1/25, *w*/*w*. An amount of 1 mL of each sample was centrifuged for 10 min with a Mini-Centrifuge (Fisher Scientific SAS, Illkirch, France). The supernatant was then recovered and distributed in 96-well microplates. Absorbance was measured at 620 and 652 nm for C-phycocyanin and 565 nm using a Multiskan GO plate reader (ThermoFisher Scientific, Villebon Coutaboeuf, France) with SkanIt RE software. The resulting solid residues were then dissolved in 1 mL of MeOH and absorbance was measured at 450, 645 and 666 nm. The mass concentration of B-phycoerythrin was obtained using the equation described by Bennett et al. [64], and the calculations for chlorophylls and carotenoids were established using the equation described by Boutin et al. [65]. Error was expressed as the standard deviation.

NaDES extracts were characterized in terms of chlorophyll and carotenoid content according to Wils et al. [14]. Briefly, 100 mg of the extract was diluted by 0.8 mL of methanol, and 290 µL of the resulting solute was used for both the carotenoid and chlorophyll titration. The absorption spectra were measured using a UV–vis microplates spectrophotometer reader (Multiskan GO, Thermo Fisher Scientific, SAS, Illkirch, France) and the carotenoid (CCa) and chlorophyll (CCh) content was then calculated using the absorbances at 450, 645 and 663 nm.

#### 3.5.2. Dye Profile

The qualitative chlorophylls/carotenoids profile was determined using high-performance liquid chromatography (HPLC) based on Jaime et al. [2]. The samples from the titration assay were then filtered through a syringe filter with a porosity of 0.45 µm and transferred into HPLC vials. The samples were analyzed on a Dionex U3000RS HPLC chain equipped with a diode array (Thermo Fisher Scientific SAS). An amount of 5 µL of each sample was then injected into a column (Accucore aQ 150 mm × 3 mm × 2.6 µm) accompanied by a precolumn (13 mm × 0.3 mm) (Thermo Fisher Scientific SAS). The flow was set at 0.8 mL/min and the column temperature was maintained at 30 °C. The mobile phases were (A) MeOH/ammonium acetate 0.1 M (aq) (7:3, *v*/*v*) and (B) MeOH 100%. The gradient was set as follow: initial solvent B content was 25%, raised to 50% in 0.64 min, then 100% in 6.6 min, and maintained for 17 min.

#### 3.5.3. FFA Profile

The FFA content of the biomass was determined using liquid chromatography-mass spectroscopy (LC-ESI-MS), according to Wils et al. [3], with no modifications. Briefly, LC-ESI-MS analyses were performed on an Acquity H-Class with a SQD detector (Waters, Saint Quentin en Yvelines, France). The system was fitted with a BEH C18 (50 mm × 2.1 mm; 1.7 µm particle size). The column oven was set at 60 °C. The mobile phases were (A) 0.01% formic acid (aq) containing 0.2 mM ammonium formate and (B) 50% isopropanol in acetonitrile containing 0.01% formic acid (aq) and 0.2 mM ammonium formate. The flow rate was 0.24 mL min−1 and the gradient was set as follows: initial solvent B content was 50%, raised to 98% in 16 min, and maintained for 4 min. The column was then re-equilibrated to initial conditions. ESI in negative mode was performed with cone voltage set at 45 V and capillary voltage at 3.5 kV. NaDES extracts required a pretreatment prior to LC-ESI-MS analyses. Solid-phase extraction was performed using C18-silica cartridges (HyperSep 1 g, 40–60 µm, Fischer Scientific, Waltham, MA, USA) with an elution gradient of different mixtures of water and MeOH ranging from 50 to 100% of MeOH. The 100% methanolic fractions were analyzed.

### 3.6. Biological Evaluation

#### 3.6.1. Sample Preparation

Extracts based on polar NADES were re-suspended in phosphate-buffered saline solution (PBS, Gibco) and extracts from non-polar NADES were solubilized in 5% DMSO final solution.

#### 3.6.2. Determination of Keratinocyte Viability

The human keratinocyte line HaCaT was cultured in 96-well plates at 4 × 10^4^ cells per well in 0.1 mL Dulbecco’s modified Eagle’s minimal essential medium (Gibco, Carlsbad, CA, USA) supplemented with 10% FBS and until approximately 80% confluence before being treated by various concentrations of NADES and NADES extracts for 24 h. Keratinocyte viability was assessed using the cell proliferation kit II (XTT) and the cytotoxicity detection kit (LDH) (Roche Diagnostics GmbH, Mannheim, Germany) according to the manufacturer’s protocols [14]. 

#### 3.6.3. Determination of Bacterial Viability

Four Gram-positive bacterial strains were used: *Staphylococcus aureus* ATCC 29213, *Staphylococcus epidermidis* CCM 2124, *Corynebacterium xerosis* ATCC 373, and *Cutibacterium acnes* ATCC 6919. *S. epidermidis* and *S. aureus* strains were cultured on Mueller–Hinton (MH; Oxoid) agar plates incubated for 24 h at 37 °C in aerobic atmospheres. *C. xerosis* and *C. acnes* strains were cultured on MH agar plates supplemented with 5% defibrinated horse blood (Oxoid) at 37 °C for 72 h in aerobic and anaerobic atmospheres, respectively. The effect of NADES and NADES extracts on bacteria viability was investigated using the tetrazolium sodium XTT reduction assay (cell proliferation kit II, Roche). For the XTT assay, bacteria were suspended in PBS containing 10% brain–heart infusion (BHI) medium and used at final bacterial concentrations of 10^6^ colony forming units (CFU)/mL for *S. aureus* and *C. xerosis*, 10^7^ CFU/mL for *S. epidermidis*, and 10^9^ CFU/mL for *C. acnes*. Bacterial concentrations were determined by measuring the optical density (OD) of the suspension at 600 nm and by CFU counts for each strain, controlled by serial 10-fold dilutions of the bacterial suspensions and plating on MH or blood-supplemented MH agar plates depending on the species. Bacteria in the presence or absence of NADES and NADES extracts at various concentrations were incubated for 4 h at 37 °C prior to adding the XTT/menadione reagent. After 2 h at 37 °C, the absorbance at 490 nm with a wavelength correction at 620 nm was read using a microplate reader (Tecan Infinite F50, Tecan) [14].

### 3.7. Statistical Analysis

The results were analyzed by GraphPad Prism version 5 (GraphPad Software, La Jolla, CA, USA). The statistical significance was evaluated by a one-way ANOVA followed by a Tuckey test performed for pigment extraction performance comparison, and a one-way ANOVA and a Kruskall–Wallis test followed by Dunn’s comparison test were applied for biological evaluation. Differences were significant at *p* < 0.05. 

## 4. Conclusions

This study demonstrated the significance of alkanediol-based NaDES for the valorization of spirulina and *P. cruentum* cakes. Novel NaDES enabled the production of FFA profiles enriched in PUFA of dermatological interest. The utilization of the innovative DAC extraction process represents a notable advancement in terms of the recovery of these PUFA. Indeed, at high speed, this process made it possible to increase the recovery of FFA by a minimum factor of 10 and to promote the preservation of PUFA by working at room temperature. The enriched extracts obtained as well as the new NaDES based on alkanediols showed increased tolerance on keratinocyte cultures compared to other HDES described in the literature. Furthermore, these NaDES and extracts demonstrated dose-dependent cytotoxic effects on two strains of staphylococci while preserving commensal species, an effect which can be due to the metabolites present in the cakes, particularly PUFA. This work highlights the relevance of the combined use of intrinsically active alkanediol-based NaDES and biomass extracts for alternative approaches to the management of skin dysbiosis. 

## Figures and Tables

**Table 1:** Alkanediol-based NaDES composition and physico-chemical properties.

NaDES Code	Component 1	Component 2	Component 3	Molar Ratio (Water%)	Heating * (°C)	Density (g/mL)	Water Miscibility
PC8	1,3-propanediol	Octanoic acid	-	1:5	50	0.914	−
PM		L-Menthol	-	1:1	60	0.946	−
PG		Glycerol	-	1:1	60	1.135	+
PCit		Citric acid	-	1:1	80	1.272	+
PLac		Lactic acid	-	1:1	50	1.109	+
PLev		Levulinic acid	-	1:1	50	1.095	+
PP		Proline	-	8:1	60	1.072	+
PGC8		Glycerol	Octanoic acid	2:1:1	50	1.028	−
PLevC8		Levulinic acid	Octanoic acid	2:1:1	50	1.018	−
PC8C10		Octanoic acid	Decanoic acid	1:3:1	20	0.912	−
OC8	1,2-octanediol	Octanoic acid	-	1:5	50	0.901	−
OM		L-Menthol	-	1:1	60	0.886	−
OG		Glycerol	-	1:1	60	1.010	−
OCit		Citric acid	Water	1:1 (20%)	80	1.161	+
OLac		Lactic acid	-	1:1	60	0.975	+
OLev		Levulinic acid	-	1:1	60	0.980	+
OP		Proline	-	8:1	60	0.941	−
OGC8		Glycerol	Octanoic acid	2:1:1	50	0.967	−
OLevC8		Levulinic acid	Octanoic acid	2:1:1	50	0.956	−
OC8C10		Octanoic acid	Decanoic acid	1:1:2	50	0.900	−

* heating temperature used for NaDES synthesis.

**Table 2:** Cake characteristics (dry matter, pigment, and lipid content). Data represent the mean ± SD. DM = dry matter.

Microalgae	Code	DM (%)	Phycobiliprotein (mg/g DM) *n* = 3	Chlorophylls (mg/g DM) *n* = 3	Carotenoids (mg/g DM) *n* = 3
*A. platensis*	Sp-Cake	17.2	84.8 ± 0.4	4.4 ± 0.1	1.6 ± 0.1
*P. cruentum*	Pc-Cake	9.0	12.9 ± 0.2	1.9 ± 0.1	0.8 ± 0.1

## Data Availability

Raw datasets generated during this study are available from the corresponding authors upon reasonable request.
